# Supplementary material for: Mechanisms governing the pioneering and redistribution capabilities of the non-classical pioneer PU.1
Source: Nat Commun. 2020 Jan 21;11:402. doi: 10.1038/s41467-019-13960-2 (PMC6972792; doi:10.1038/s41467-019-13960-2)
Supplement: Supplementary file 7 — Source data [file 41467_2019_13960_MOESM7_ESM.zip › Source_Data/FigureS2/FigureS2B_MotifScanOutput/KmeansCluster11/homerResults.html]

/misc/data/analysis/project\_PU1/CTV1/motifs/basic/PU1\_Kmeans14.11/final - Homer de novo Motif Results


# Homer *de novo* Motif Results (/misc/data/analysis/project\_PU1/CTV1/motifs/basic/PU1\_Kmeans14.11/final)

Known Motif Enrichment Results  
Gene Ontology Enrichment Results  
If Homer is having trouble matching a motif to a known motif, try copy/pasting the matrix file into
STAMP  
More information on motif finding results: HOMER
| Description of Results
| Tips
  
Total target sequences = 3249  
Total background sequences = 45230  
\* - possible false positive  

|  |  |  |  |  |  |  |  |  |
| --- | --- | --- | --- | --- | --- | --- | --- | --- |
| Rank | Motif | P-value | log P-pvalue | % of Targets | % of Background | STD(Bg STD) | Best Match/Details | Motif File |
| 1 | T C A G C G T A T A C G T C G A C T A G T C A G G C T A C G T A T A C G G A C T T A C G | 1e-1277 | -2.941e+03 | 82.73% | 17.98% | 34.4bp (68.2bp) | PU.1(ETS)/ThioMac-PU.1-ChIP-Seq(GSE21512)/Homer(0.966) More Information \| Similar Motifs Found | motif file (matrix) |
| 2 | C T A G A T C G A C G T A C G T A G T C G A T C A T C G A G C T C A G T A C G T A G C T | 1e-174 | -4.009e+02 | 21.91% | 6.11% | 46.5bp (67.3bp) | SpiB(ETS)/OCILY3-SPIB-ChIP-Seq(GSE56857)/Homer(0.834) More Information \| Similar Motifs Found | motif file (matrix) |
| 3 | A T C G G C A T C T G A T C G A A G T C A T G C C T G A T G A C C T G A C T G A | 1e-140 | -3.239e+02 | 28.32% | 11.27% | 52.0bp (62.0bp) | RUNX(Runt)/HPC7-Runx1-ChIP-Seq(GSE22178)/Homer(0.973) More Information \| Similar Motifs Found | motif file (matrix) |
| 4 | T G A C C T G A A T G C A G C T C G A T T G A C G A T C A G C T A C G T A T G C A G T C | 1e-138 | -3.192e+02 | 15.48% | 3.77% | 51.5bp (70.4bp) | RBM5(Znf)/Homo\_sapiens-RNCMPT00055-PBM/HughesRNA(0.790) More Information \| Similar Motifs Found | motif file (matrix) |
| 5 | T A C G A G C T C A T G T G A C A G T C G T A C A G T C A G T C G A C T C T G A A T C G C G A T A C T G T A C G | 1e-92 | -2.131e+02 | 5.11% | 0.46% | 53.4bp (63.6bp) | BORIS(Zf)/K562-CTCFL-ChIP-Seq(GSE32465)/Homer(0.954) More Information \| Similar Motifs Found | motif file (matrix) |
| 6 | A G T C G T A C C T A G T A C G G T C A C G T A T A C G C A G T C A T G A C T G A T G C A G C T | 1e-91 | -2.114e+02 | 6.80% | 1.00% | 47.5bp (59.2bp) | Gabpa/MA0062.2/Jaspar(0.870) More Information \| Similar Motifs Found | motif file (matrix) |
| 7 | A T G C G A T C C G A T A G T C A G T C A G T C T G A C T G A C A G C T | 1e-62 | -1.428e+02 | 31.43% | 18.70% | 54.4bp (66.9bp) | SRSF1(RRM)/Homo\_sapiens-RNCMPT00163-PBM/HughesRNA(0.849) More Information \| Similar Motifs Found | motif file (matrix) |
| 8 | T G C A A T G C G A C T C G A T T A G C T A G C A C G T C G T A A T C G C G A T | 1e-57 | -1.333e+02 | 10.53% | 3.70% | 56.5bp (67.4bp) | ELF3(ETS)/PDAC-ELF3-ChIP-Seq(GSE64557)/Homer(0.812) More Information \| Similar Motifs Found | motif file (matrix) |
| 9 | A G C T C G T A T A G C A G C T A G C T A T G C A G T C A C G T C G A T A C G T G T A C A C G T | 1e-57 | -1.328e+02 | 3.60% | 0.41% | 50.2bp (54.6bp) | Eip74EF/dmmpmm(Bigfoot)/fly(0.730) More Information \| Similar Motifs Found | motif file (matrix) |
| 10 | C G T A C G T A A G T C A G T C A C T G G C T A G T C A | 1e-46 | -1.076e+02 | 50.72% | 37.79% | 57.1bp (65.7bp) | MYB81(MYB)/col-MYB81-DAP-Seq(GSE60143)/Homer(0.939) More Information \| Similar Motifs Found | motif file (matrix) |
| 11 | T G C A C A G T A T C G G T C A C A T G A G C T T A G C T C G A A G C T | 1e-42 | -9.750e+01 | 9.45% | 3.76% | 55.4bp (61.6bp) | JDP2/MA0655.1/Jaspar(0.965) More Information \| Similar Motifs Found | motif file (matrix) |
| 12 | C T A G A G T C C G A T A C T G G C T A A C G T C T A G G T C A | 1e-39 | -9.063e+01 | 47.55% | 35.81% | 57.2bp (64.7bp) | Unknown5/Drosophila-Promoters/Homer(0.735) More Information \| Similar Motifs Found | motif file (matrix) |
| 13 | A G T C A T G C C A G T A C G T G C A T A C T G G C T A G C A T T A C G A G C T | 1e-34 | -8.039e+01 | 9.05% | 3.90% | 56.1bp (64.0bp) | TCF7L2/MA0523.1/Jaspar(0.918) More Information \| Similar Motifs Found | motif file (matrix) |
| 14 | G T C A A G T C T G C A A G C T G T A C G T A C A C G T T C A G A G C T G A C T | 1e-32 | -7.372e+01 | 2.92% | 0.56% | 56.8bp (65.2bp) | SPDEF(ETS)/VCaP-SPDEF-ChIP-Seq(SRA014231)/Homer(0.841) More Information \| Similar Motifs Found | motif file (matrix) |
| 15 | C T A G A C T G T C A G C T G A C A T G C T A G T C A G T C G A A C T G C T G A C A T G A C T G C G T A | 1e-31 | -7.344e+01 | 14.59% | 8.07% | 58.2bp (69.9bp) | SRSF1(RRM)/Homo\_sapiens-RNCMPT00163-PBM/HughesRNA(0.719) More Information \| Similar Motifs Found | motif file (matrix) |
| 16 | G A C T A G C T A T G C G T A C A C G T G A T C G T A C G T A C T A G C G T A C C T G A A T G C C G T A A C T G | 1e-28 | -6.586e+01 | 2.99% | 0.66% | 52.8bp (65.5bp) | Zfp281(Zf)/ES-Zfp281-ChIP-Seq(GSE81042)/Homer(0.768) More Information \| Similar Motifs Found | motif file (matrix) |
| 17 | G A T C G C T A C T A G C T A G T C G A C T G A T C A G C A G T C T A G A T G C C A G T A C T G | 1e-27 | -6.345e+01 | 6.28% | 2.52% | 51.7bp (61.5bp) | ERG(ETS)/VCaP-ERG-ChIP-Seq(GSE14097)/Homer(0.712) More Information \| Similar Motifs Found | motif file (matrix) |
| 18 | A T G C G C T A T A G C A C G T A C G T A G T C A C G T A T C G A G T C G C A T G C A T | 1e-25 | -5.971e+01 | 3.91% | 1.20% | 40.4bp (67.3bp) | SpiB(ETS)/OCILY3-SPIB-ChIP-Seq(GSE56857)/Homer(0.771) More Information \| Similar Motifs Found | motif file (matrix) |
| 19 | C A T G C G A T A C T G A T C G C G A T A C G T G A T C A G T C C G A T A T C G G C A T A C G T C G A T | 1e-22 | -5.143e+01 | 1.82% | 0.31% | 49.8bp (69.6bp) | Ets1-distal(ETS)/CD4+-PolII-ChIP-Seq(Barski\_et\_al.)/Homer(0.693) More Information \| Similar Motifs Found | motif file (matrix) |
| 20 | A C G T A C T G A T C G A C G T A C G T C A G T A T C G C G T A C A G T C T A G C G A T C T A G C T A G | 1e-22 | -5.120e+01 | 3.11% | 0.90% | 53.3bp (58.5bp) | UNC-75(RRM)/Caenorhabditis\_elegans-RNCMPT00081-PBM/HughesRNA(0.679) More Information \| Similar Motifs Found | motif file (matrix) |
| 21 | C T A G A T G C G A T C A G T C A G T C A G T C A G T C C A G T A G T C G T A C G A T C A G C T A T G C | 1e-22 | -5.083e+01 | 6.99% | 3.31% | 55.4bp (65.9bp) | RBM5(Znf)/Homo\_sapiens-RNCMPT00055-PBM/HughesRNA(0.717) More Information \| Similar Motifs Found | motif file (matrix) |
| 22 | G A T C T G C A C G T A G C A T G C T A T A G C G A T C C T A G G T A C | 1e-21 | -5.013e+01 | 7.73% | 3.85% | 59.1bp (62.1bp) | PH0158.1\_Rhox11\_2/Jaspar(0.640) More Information \| Similar Motifs Found | motif file (matrix) |
| 23 | A C T G T C A G T C G A C T G A A C T G C A G T C T A G C A T G T C A G C G A T A C T G C T A G T C A G A C T G | 1e-21 | -4.987e+01 | 2.52% | 0.63% | 51.7bp (64.7bp) | KLF5/MA0599.1/Jaspar(0.721) More Information \| Similar Motifs Found | motif file (matrix) |
| 24 | C G T A A C G T A C G T A C G T A G T C A G T C A G T C | 1e-20 | -4.737e+01 | 11.67% | 6.90% | 52.0bp (65.7bp) | Pcbp2(KH)/Danio\_rerio-RNCMPT00246-PBM/HughesRNA(0.868) More Information \| Similar Motifs Found | motif file (matrix) |
| 25 | A C G T T G A C C A T G A G T C G A C T C G A T A G C T A T C G C T G A G C A T | 1e-20 | -4.617e+01 | 4.03% | 1.51% | 58.4bp (60.4bp) | pan/dmmpmm(Papatsenko)/fly(0.792) More Information \| Similar Motifs Found | motif file (matrix) |
| 26 | A T C G T A G C A T G C T G A C A G C T C G A T A G T C G A T C A C G T G T A C G T A C | 1e-19 | -4.598e+01 | 13.82% | 8.68% | 56.7bp (65.4bp) | NCU02404(RRM)/Neurospora\_crassa-RNCMPT00238-PBM/HughesRNA(0.760) More Information \| Similar Motifs Found | motif file (matrix) |
| 27 | G A T C A G T C A G C T A G C T G A T C G T A C A T G C G C A T A T C G T G A C G A T C T A G C G C A T | 1e-19 | -4.524e+01 | 17.48% | 11.75% | 53.4bp (66.5bp) | PCBP2(KH)/Homo\_sapiens-RNCMPT00044-PBM/HughesRNA(0.629) More Information \| Similar Motifs Found | motif file (matrix) |
| 28 | G T A C C T A G G T A C A T G C A G T C G T C A T A G C C T G A | 1e-19 | -4.482e+01 | 18.04% | 12.24% | 54.3bp (63.7bp) | Egr2(Zf)/Thymocytes-Egr2-ChIP-Seq(GSE34254)/Homer(0.852) More Information \| Similar Motifs Found | motif file (matrix) |
| 29 | A C G T G C A T T A C G A C T G A C G T A C G T G A C T G T A C G T A C C T A G G A T C | 1e-19 | -4.400e+01 | 3.17% | 1.05% | 58.1bp (63.0bp) | che-1/MA0260.1/Jaspar(0.740) More Information \| Similar Motifs Found | motif file (matrix) |
| 30 | G A T C A C T G C G A T A G T C C G T A A C T G A C G T C G T A | 1e-18 | -4.333e+01 | 7.91% | 4.22% | 56.8bp (64.5bp) | SUM1(MacIsaac)/Yeast(0.812) More Information \| Similar Motifs Found | motif file (matrix) |
| 31 | C A T G T C G A T A C G C A T G C T A G A T G C C T A G | 1e-18 | -4.329e+01 | 37.06% | 29.45% | 56.8bp (65.8bp) | btd/dmmpmm(Noyes)/fly(0.767) More Information \| Similar Motifs Found | motif file (matrix) |
| 32 | A C G T A C G T C G T A A C G T A G T C A C G T A T G C | 1e-18 | -4.152e+01 | 11.73% | 7.23% | 55.9bp (63.0bp) | GATA3(Zf)/iTreg-Gata3-ChIP-Seq(GSE20898)/Homer(0.925) More Information \| Similar Motifs Found | motif file (matrix) |
| 33 | A C G T C A G T C G A T C A T G A G T C C T G A C G A T G C T A C G T A | 1e-16 | -3.858e+01 | 10.03% | 6.03% | 60.2bp (62.2bp) | Oct4(POU,Homeobox)/mES-Oct4-ChIP-Seq(GSE11431)/Homer(0.895) More Information \| Similar Motifs Found | motif file (matrix) |
| 34 | C A G T A C T G C T A G A G T C C G A T A C G T A G C T A C G T A C T G C A G T A C T G T A C G A C G T A C T G | 1e-16 | -3.738e+01 | 0.71% | 0.04% | 69.2bp (60.2bp) | Sf3b4(RRM)/Danio\_rerio-RNCMPT00224-PBM/HughesRNA(0.695) More Information \| Similar Motifs Found | motif file (matrix) |
| 35 | A C T G A C G T C A T G G T C A A T G C A C G T T A G C G T C A A T G C C G T A | 1e-15 | -3.583e+01 | 4.37% | 1.97% | 54.5bp (62.0bp) | Pknox1(Homeobox)/ES-Prep1-ChIP-Seq(GSE63282)/Homer(0.806) More Information \| Similar Motifs Found | motif file (matrix) |
| 36 | C T G A T C A G T A G C G C T A T A C G T G A C C G T A C T A G T A G C C G T A A T C G G T A C C G A T T C A G | 1e-14 | -3.420e+01 | 22.04% | 16.47% | 55.9bp (64.4bp) | PB0003.1\_Ascl2\_1/Jaspar(0.794) More Information \| Similar Motifs Found | motif file (matrix) |
| 37 | A T G C C G T A T A G C G C A T A C T G A T G C A G C T C G A T A G T C A G C T A G T C A C G T | 1e-13 | -3.214e+01 | 3.17% | 1.28% | 53.1bp (63.8bp) | SRSF10(RRM)/Homo\_sapiens-RNCMPT00088-PBM/HughesRNA(0.704) More Information \| Similar Motifs Found | motif file (matrix) |
| 38 | T C G A A G T C G T C A A G C T A C G T A C T G G T A C A C T G A C T G A G C T C A G T A C G T A G C T | 1e-13 | -3.181e+01 | 0.65% | 0.05% | 59.4bp (56.4bp) | RUNX2/MA0511.2/Jaspar(0.755) More Information \| Similar Motifs Found | motif file (matrix) |
| 39 | A G T C C G A T G C A T C T G A A T G C T G C A A C T G A G T C | 1e-13 | -3.030e+01 | 14.40% | 10.10% | 57.3bp (65.1bp) | DEL2(E2FDP)/col-DEL2-DAP-Seq(GSE60143)/Homer(0.760) More Information \| Similar Motifs Found | motif file (matrix) |
| 40 | T A G C G T C A A C G T A C G T C G T A A G T C G T C A A G T C G T C A | 1e-13 | -3.021e+01 | 4.03% | 1.90% | 56.9bp (61.1bp) | slbo/dmmpmm(Pollard)/fly(0.802) More Information \| Similar Motifs Found | motif file (matrix) |
| 41 | G T C A A C T G C T A G G T C A G T C A T A C G C T G A C A T G C T A G G T C A C A T G A C T G G C T A T G C A | 1e-12 | -2.963e+01 | 1.69% | 0.48% | 58.9bp (73.9bp) | SF2(RRM)/Drosophila\_melanogaster-RNCMPT00066-PBM/HughesRNA(0.678) More Information \| Similar Motifs Found | motif file (matrix) |
| 42 | A T G C C T G A A C G T A T C G G T A C C T A G A T C G C G T A C G T A A G T C C G T A A C T G G A T C T A G C | 1e-12 | -2.917e+01 | 0.71% | 0.07% | 47.6bp (55.1bp) | ZNF189(Zf)/HEK293-ZNF189.GFP-ChIP-Seq(GSE58341)/Homer(0.702) More Information \| Similar Motifs Found | motif file (matrix) |
| 43 | G T C A A C T G G A C T A C G T G A T C G T A C T A G C G A T C G C A T A C T G G A C T T A G C | 1e-12 | -2.899e+01 | 1.35% | 0.32% | 45.3bp (66.0bp) | RO3G\_00049(RRM)/Rhizopus\_oryzae-RNCMPT00205-PBM/HughesRNA(0.797) More Information \| Similar Motifs Found | motif file (matrix) |
| 44 | T C G A A G C T C T A G A G T C A G T C A C G T G C A T A G T C G T A C A G C T A T G C | 1e-12 | -2.818e+01 | 1.97% | 0.65% | 54.2bp (60.2bp) | GCR1/Literature(Harbison)/Yeast(0.746) More Information \| Similar Motifs Found | motif file (matrix) |
